# Off-Label Use of Bevacizumab in Patients Diagnosed with Age-Related Macular Degeneration: A Systematic Review and Meta-Analysis

**DOI:** 10.3390/ph17081000

**Published:** 2024-07-29

**Authors:** João Estarreja, Priscila Mendes, Carina Silva, Pedro Camacho, Vanessa Mateus

**Affiliations:** 1 H&TRC—Health and Technology Research Center, ESTeSL—Escola Superior de Tecnologia da Saúde, Instituto Politécnico de Lisboa, 1990-096 Lisbon, Portugal; joaoestarreja@edu.ulisboa.pt (J.E.); priscila.mendes@estesl.ipl.pt (P.M.); carina.silva@estesl.ipl.pt (C.S.); pedro.camacho@estesl.ipl.pt (P.C.); 2 Centro de Estatística e Aplicações, Universidade de Lisboa, 1749-016 Lisbon, Portugal; 3 Research Institute for Medicines (iMed.ULisboa), Faculty of Pharmacy, Universidade de Lisboa, Av. Professor Gama Pinto, 1649-003 Lisbon, Portugal

**Keywords:** AMD, age-related macular degeneration, bevacizumab, drug therapy, nAMD, neovascular age-related macular degeneration, wet macular degeneration

## Abstract

Background: Age-related macular degeneration (AMD) is the leading cause of vision loss in elderly people. Current pharmacological treatment in vascular AMD includes anti-VEGF agents, such as ranibizumab and aflibercept. Additionally, the off-label use of bevacizumab has been shown to be effective and has a lower cost, making it an interesting pharmacological approach; however, there is no consensus about its use. Therefore, this systematic review and meta-analysis aims to evaluate the efficacy, safety, and efficiency of bevacizumab in AMD patients. Methods: This review only focused on randomized controlled clinical trials published in 2010 in the MEDLINE database that compared the effect of bevacizumab with ranibizumab. The risk of bias in each included study was assessed using the CASP Randomised Clinical Trials checklist. Results: Twelve studies were included for qualitative synthesis, and nine of them were considered for meta-analysis. Bevacizumab-treated patients showed a significantly reduced neovascularization in a longer spectrum of time; however, they had a higher incidence of endophthalmitis than those treated with ranibizumab. Regarding efficiency, the mean number of administrations was reduced in the treatment with bevacizumab in comparison to ranibizumab. Conclusions: Clinical evidence demonstrates that bevacizumab has efficacy and safety profiles comparable with ranibizumab; however, it is relatively more efficient.

## 1. Introduction

Age-related macular degeneration (AMD) is recognized as a multifactorial disease, being the leading cause of vision loss in individuals aged 55 years and older [1,2,3]. Considering its high prevalence and trend of new cases in the future, which can be associated with the phenomenon of aging, this disorder may affect around 290 million people in the next two decades [1,4]. Currently, AMD presents considerable healthcare costs, emphasizing its significant socioeconomic impact worldwide [5].

AMD can be categorized into three stages, namely early, intermediate, and late [6]. In a general mode, 90% of the cases of AMD are diagnosed in the early and intermediate stages, usually characterized as asymptomatic [6,7,8]. However, the late stages, namely non-vascular (atrophic) AMD and neovascular AMD (nAMD), are the main causes of irreversible vision loss [4,5].

AMD is usually initiated by the presence of drusen and hyperpigmentation, which can be accompanied by retinal pigment epithelium (RPE) dysfunction, increasing the risk of geographic atrophy or nAMD [6,9,10]. Indeed, nAMD is normally associated with the loss of choroidal vasculature, being sustained by a reduction in blood supply due to stenosis of larger vessels [6,9]. In normal conditions, RPE persists intact; however, considering the reduced blood supply, it becomes hypoxic and releases angiogenic substances, such as vascular endothelial growth factor (VEGF) [9]. Therefore, it promotes the formation of vessels from choriocapillaris, being recognized as choroidal neovascularization [9].

In the last 20 years, the intravitreal administration of anti-VEGF (aVEGF) drugs (e.g., aflibercept, ranibizumab (RBZ), bevacizumab (BVZ), and brolucizumab) has been the standard of care for the pharmacological treatment of nAMD, where the most commonly used is RBZ [11,12,13,14,15]. In a general mode, the number of injections and repeated treatments with aVEGF agents represent a tremendous burden for elderly patients. Moreover, almost 50% of nAMD patients do not have an optimal and sustained response to these drugs, highlighting the need to investigate new longer, longer-acting pharmacological approaches with higher effectiveness over time [15,16].

BVZ, the most popular aVEGF drug, originally approved for the treatment of metastatic colorectal, breast, and lung cancers, has shown comparable efficacy to other aVEGF agents in the context of nAMD and has a significantly lower cost [11,12,17,18,19,20,21]. Indeed, several clinical studies show the effective role of BVZ in nAMD patients, and it is less expensive than other aVEGF drugs, such as RBZ and aflibercept [12,17,22]. However, due to the existence of medicines already approved for the treatment of nAMD by the respective regulatory authorities, along with controversial results obtained in several studies, at the moment, there is no consensus in the medical community of prescribing BVZ to the detriment of other drugs. Another important aspect is related to the therapeutic regimens usually described in clinical trials, which are difficult to maintain in a real-world scenario. In fact, the adaptation of different treatment schemes has been observed, considering, for instance, the pro re nata approach, where patients are treated by necessity and not by a continuous design. Since this approach is normally associated with low levels of effectiveness, a treat-and-extend regimen has been suggested; however, it presents some disadvantages, such as high costs and reduced patient compliance [14].

According to the literature, reviews are already discussing the efficacy and safety of BVZ in nAMD, along with its comparison to other pharmacological agents (e.g., RBZ and aflibercept) [23,24,25]. Nevertheless, there is a noticeable lack of evidence regarding its efficiency (e.g., the number of intravitreal injections needed to obtain a sustained pharmacological response). Indeed, the analysis of the efficiency demonstrated by BVZ, in comparison to other aVEGF drugs, may be essential to decision-making since it would reduce the number of intraocular administrations performed in patients, increasing their compliance and overall quality of life. Additionally, regarding the expiration of the patents in some aVEGFs, the prices will be potentially reduced in the future, making it important to compare two of the most commonly used aVEGFs available on the market, namely BVZ and RBZ [26]. Therefore, this systematic review and meta-analysis aimed to evaluate the efficacy, safety, and efficiency of BVZ in the treatment of patients aged 50 years and older diagnosed with nAMD in comparison to RBZ through the analysis of randomized clinical trials.

## 2. Results

### 2.1. Study Selection

**Figure 1:**
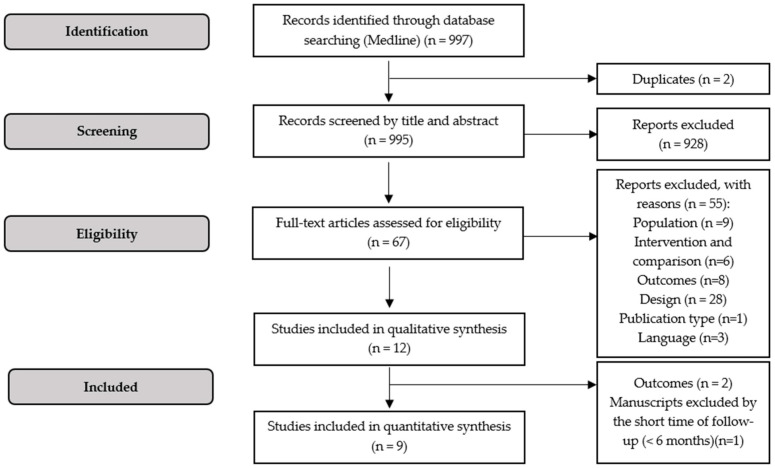
Flow diagram representing the process of study selection for qualitative and quantitative syntheses.

A total of nine hundred ninety-seven records were identified after the application of the search expression in MEDLINE via the PubMed platform, in which two of them were duplicates (Figure 1). Considering the analysis of the titles and abstracts of the 995 articles potentially eligible, 928 were excluded. Afterward, the full texts of the remaining 67 articles were carefully analyzed, resulting in the exclusion of 55 studies for the following reasons: studies that included individuals with other ocular diseases and/or aged 50 years or less (*n* = 9); intervention and comparison without interest (e.g., switching therapy) (*n* = 6); studies that focused on other outcomes without interest for this review (*n* = 8); non-randomized clinical trials (e.g., observational studies) (*n* = 28); systematic review (*n* = 1); and studies not written in English (e.g., Chinese) (*n* = 3). From the twelve remaining studies included for qualitative synthesis, three studies were not considered for the meta-analysis for the following reasons: the studies lacked adequate or sufficient outcomes (*n* = 2) and studies had a relatively short time of follow-up (e.g., <6 months) (*n* = 1).

### 2.2. Study Characteristics

A qualitative synthesis was performed using the 12 included randomized clinical trials, where relevant data were extracted regarding the basic characteristics of each one (Table 1), namely the country, sample size, age of the participants, duration of treatment, and therapeutic regimen studied. Furthermore, relevant information concerning efficacy (Table 2) and safety (Table 3) outcomes of interest was also extracted and evaluated in each study. The majority of the information presented in Table 2 and Table 3 was subjected to a quantitative synthesis; however, there were studies not included in the meta-analysis, and it is important to consider all the clinical evidence currently available.

### 2.3. Risk of Bias Assessment

Each included study was subjected to a careful risk of bias assessment, considering the methodological quality and internal validity, through the use of the Critical Appraisal Skills Programme Randomised Controlled Trials checklist (Table 4) [27]. Theoretically, a higher overall quality of grading is associated with a lower risk of bias. Regarding the results, it was not possible to identify any study with low-quality grading and a higher risk of bias consecutively. Indeed, from the twelve randomized clinical trials assessed, eight of them were qualified as high-quality, whereas the remaining four were categorized as moderate.

**Table 1:** General characteristics of each randomized clinical trial included.

Author (year)	Country	Sample (n)	Age (years)		Duration (months)	Regimen
			Bevacizumab (mean ± SD)	Ranibizumab (mean ± SD)		
Nunes et al. (2019) [28]	Brazil	44	75.60 ± 7.95	-	12	Fortnightly
			75.27 ± 8.77	75.00 ± 7.89		Monthly
Berg et al. (2016) [29]	Norway	339	78.70 ± 7.60	78.00 ± 8.20	24	Monthly to as needed
Schauwvlieghe et al. (2016) [30]	Netherlands	327	79.00 ± 7.00	78.00 ± 8.20	12	Monthly
Chakravarthy et al. (2015) [31]	United Kingdom	610	77.70 ± 7.30	77.80 ± 7.60	24	Monthly or as needed
Berg et al. (2015) [32]	Norway	371	78.70 ± 7.60	78.00 ± 8.20	12	Monthly to as needed
Sizmaz et al. (2014) [33]	Turkey	40	73.30 ± 6.30	73.20 ± 6.20	1	Single administration
Scholler et al. (2014) [34]	Austria	46	80.75 ± 6.55	79.54 ± 6.78	12	Monthly to as needed
Kodjikian et al. (2013) [35]	France	384	79.62 ± 6.90	78.68 ± 7.27	12	Monthly to as needed
Krebs et al. (2013) [36]	Austria	317	76.70 ± 7.80	77.60 ± 8.10	12	Monthly to as needed
Martin et al. (2012) [37]	United States of America	263	79.70 ± 7.50	79.50 ± 7.40	24	Monthly
		485	78.90 ± 7.40	78.3 ± 7.80		As needed
Biswas et al. (2011) [38]	India	104	64.36	63.48	18	Monthly to as needed
Subramanian et al. (2010) [39]	United States of America	22	78.00	80.00	12	Monthly to as needed

Legend: SD, standard deviation. Note: Values of standard deviation are not available in Biswas et al. (2011) [38] and Subramanian et al.’s (2010) [39] studies.

**Table 2:** Efficacy outcomes evaluated in each randomized clinical trial.

Author (year)	Regimen	ETDRS Letters		CMT (µm)		Thickness at the Fovea (µm)		Dye Leakage on FA (%events)		Fluid on OCT (%events)	
		BVZ	RBZ	BVZ	RBZ	BVZ	RBZ	BVZ	RBZ	BVZ	RBZ
Nunes et al. (2019) [28]	Fortnightly	+13.40	-	−166.72	-	-	-	-	-	-	-
	Monthly	+5.93	+12.33	−199.62	−216.00	-	-	-	-	-	-
Berg et al. (2016) [29]	Monthly to as needed	+7.40	+6.60	−113.00	−122.00	-	-	-	-	-	-
Schauwvlieghe et al. (2016) [30]	Monthly	+5.09	+6.40	−131.00	−138.00	-	-	-	-	−54.04%	−63.20%
Chakravarthy et al. (2015) [31]	Monthly or as needed	+4.10	+4.90	−94.90	−108.10	−133.80	−146.90	−53.70%	−48.69%	−36.95%	−44.76%
Berg et al. (2015) [32]	Monthly to as needed	+7.90	+8.20	−112.00	−120.00	-	-	-	-	-	-
Sizmaz et al. (2014) [33]	Single administration	-	-	−78.50	−91.50	−10.00	−3.10	-	-	-	-
Scholler et al. (2014) [34]	Monthly to as needed	+7.35	+0.60	−62.00	−115.66	-	-	-	-	-	-
Kodjikian et al. (2013) [35]	Monthly to as needed	+4.82	+2.93	−94.96	−107.23	-	-	+16.23%	+15.31%	−45.54	−53.56%
Krebs et al. (2013) [36]	Monthly to as needed	+5.20	+4.30	−86.30	−89.86	-	-	-	-	-	-
Martin et al. (2012) [37]	Monthly	+7.80	+8.80	−84.00	−91.00	−180.00	−91.00	-	-	-	-
	As needed	+5.00	+6.70	−84.00	−78.00	−153.00	−166.00	-	-	-	-
Biswas et al. (2011) [38]	Monthly to as needed	+3.96	+3.56	−37.96	−44.70	-	-	-	-	-	-
Subramanian et al. (2010) [39]	Monthly to as needed	+7.96	+6.30	−50.00	−91.00	-	-	-	-	-	-

Legend: ETDRS, early treatment of diabetic retinopathy study; BVZ, bevacizumab; CMT, central macular thickness; FA, fluorescein angiography; OCT, optical coherence tomography; RBZ, ranibizumab.

**Table 3:** Safety outcomes evaluated in each randomized clinical trial.

Author (year)	Regimen	IOP (mmHg)		SBP (mmHg)		DBP (mmHg)		PVD (%events)		Partial PVD (%events)		Retinal PED (%events)	
		BVZ	RBZ	BVZ	RBZ	BVZ	RBZ	BVZ	RBZ	BVZ	RBZ	BVZ	RBZ
Nunes et al. (2019) [28]	Fortnightly	-	-	-	-	-	-	+16.41%	+21.43%	−2.56%	−21.43%	−25.13%	−14.28%
	Monthly	-	-	-	-	-	-	+15.24%	-	−12.86%	-	−30.47%	-
Berg et al. (2016) [29]	Monthly to as needed	-	-	−8.20	−5.10	−3.50	−2.20	-	-	-	-	-	-
Schauwvlieghe et al. (2016) [30]	Monthly	−0.93	−0.06	-	-	-	-	-	-	-	-	-	-
Chakravarthy et al. (2015) [31]	Monthly or as needed	-	-	−3.10	−3.80	−2.50	−2.60	-	-	-	-	-	-
Berg et al. (2015) [32]	Monthly to as needed	-	-	−5.80	−4.60	−1.30	−2.30	-	-	-	-	-	-

Legend: BVZ, bevacizumab; DBP, diastolic blood pressure; IOP, intraocular pressure; PED, pigment epithelial detachment; PVD, posterior vitreous detachment; RBZ, ranibizumab; SBP, systolic blood pressure.

**Table 4:** Risk of bias assessment (methodological quality) of each included randomized clinical trial.

Author (year)	Overall Quality Grading	
	Quantitative	Qualitative
Nunes et al. (2019) [28]	17	B
Berg et al. (2016) [29]	21	A
Schauwvlieghe et al. (2016) [30]	22	A
Chakravarthy et al. (2015) [31]	18	A
Berg et al. (2015) [32]	21	A
Sizmaz et al. (2014) [33]	15	B
Scholler et al. (2014) [34]	17	B
Kodjikian et al. (2013) [35]	21	A
Krebs et al. (2013) [36]	22	A
Martin et al. (2012) [37]	19	A
Biswas et al. (2011) [38]	19	A
Subramanian et al. (2010) [39]	16	B

Note: [0–8]—low quality score (C); [9–17]—moderate quality score (B); [18–26]—high quality score (A).

### 2.4. Meta-Analysis

The results of the meta-analysis are presented for each parameter of the outcomes in the order described above, and the Supplementary Materials is organized in the same way.

Meta-analysis was initially conducted considering all available studies, and when justified, subgroup analyses are presented.

#### 2.4.1. Efficacy Outcomes

**Figure 2:**
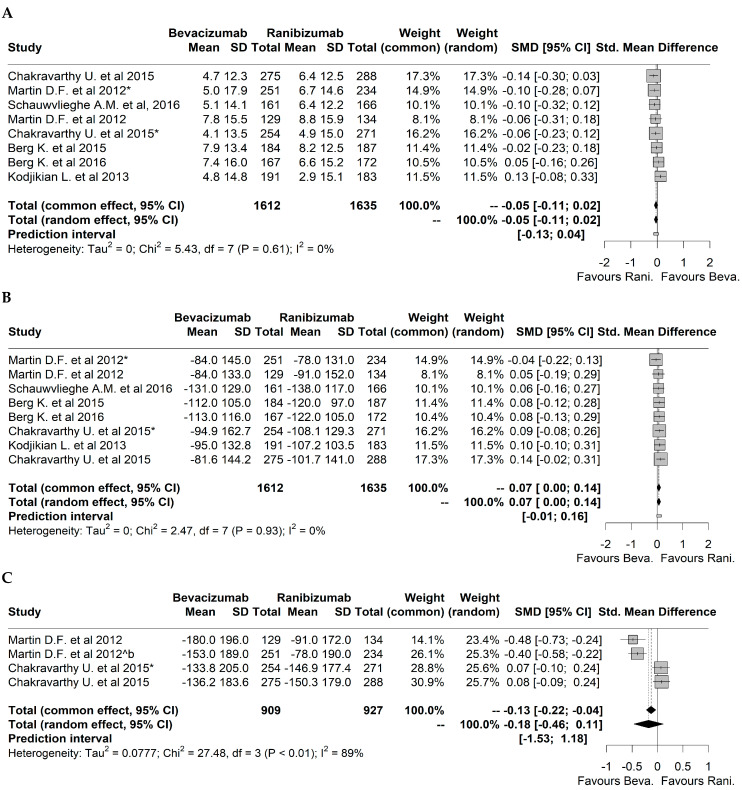
Forest plots representing the efficacy outcomes regarding treatments with bevacizumab and ranibizumab. Legend: (**A**) visual acuity; (**B**) retinal thickness; (**C**) fovea thickness. Legend: Chakravarthy et al. (2015) [31] study was analyzed considering both 1-year and 2-year (*) treatment outcomes. Martin et al. (2012) [37] study was evaluated considering both monthly and “as needed” (*) treatment protocols [29,30,31,32,35,37].

**Figure 3:**
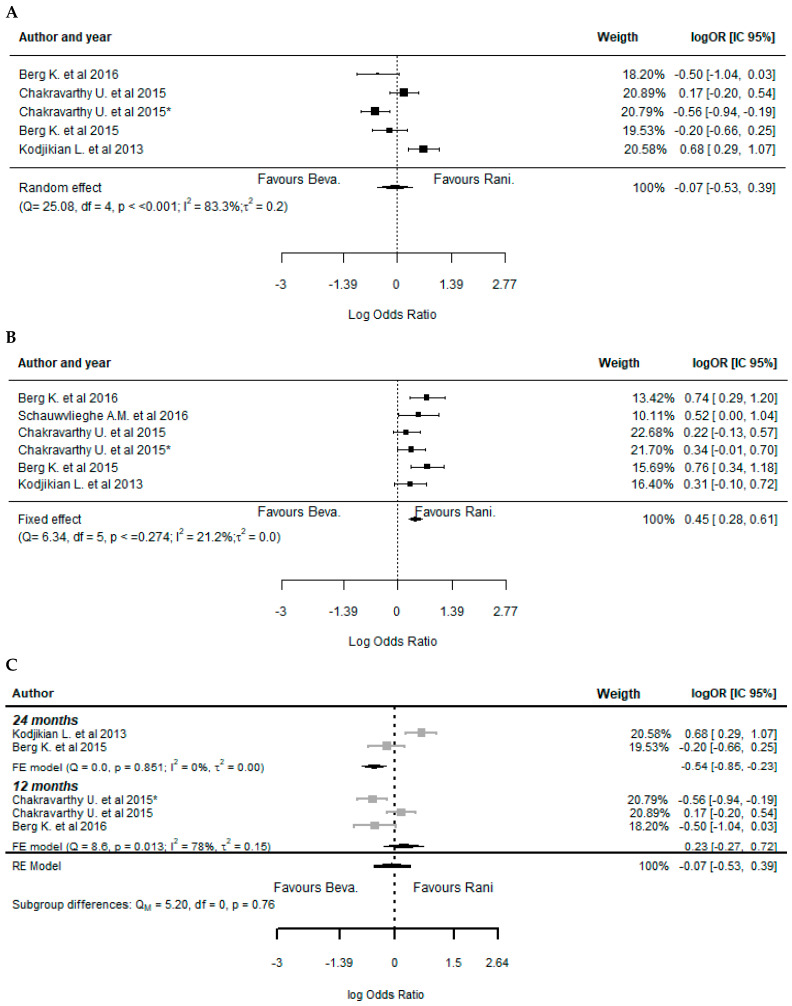
Forest plot representing efficacy outcomes of neovascularization regarding the treatment with bevacizumab and ranibizumab. Legend: (**A**), fluid on OCT; (**B**), dye leakage in FA; (**C**), subgroup analysis for dye leakage in FA. Chakravarthy et al. (2015) [31] study was analyzed considering both 1-year and 2-year (*) treatment outcomes [29,30,31,32,35].

The analysis of the efficacy outcomes in the eligible studies for quantitative synthesis showed a lack of statistically significant differences, in most cases, between the treatment with BVZ and RBZ (Figure 2). Indeed, it was not possible to favor one of the treatments applied concerning the changes in visual acuity (Figure 2A) (−0.05, 95%CI: [−0.11; 0.02]), retinal thickness (Figure 2B) (0.07, 95%CI: [0.00; 0.14]), fovea thickness (Figure 2C) (−0.18, 95%CI: [−0.46; 0.11]), and neovascularization activity (dye leakage in fluorescein angiography (FA)) (Figure 3A) (−0.07, 95%CI: [−0.53; 0.39]). However, a significantly higher presence of fluid on optical coherence tomography (OCT) structural assessment (neovascularization activity) was observed in RBZ-treated patients, which favored the treatment with BVZ (Figure 3B) (0.45, 95%CI: [0.28; 0.61]).

Since variability among studies was identified for neovascularization, represented by dye leakage in FA, a forest plot analysis was conducted, considering the subgroups of 12 months and 24 months of treatment (Figure 3C). Treatment with BVZ is favored for the subgroup of 24 months of treatment (−0.54, 95%CI: [−0.85; −0.23]), and no significant differences between treatments were found in the subgroup of 12 months of treatment (0.23, 95%CI: [−0.27; 0.72]). Overall, no significant differences between subgroups were found (*p* = 0.76).

Concerning the analysis of the publication bias among the studies analyzed, it was possible to identify sufficient evidence in some of the efficacy outcomes considered. Indeed, there was evidence for publication bias considering changes in visual acuity (Figure S1A, Egger’s test: t = 1.16, *p* = 0.292), retinal thickness (Figure S1B, Egger’s test: t = −0.382, *p* = 0.715), and fovea thickness (Figure S1C, Egger’s test: t = −1.49, *p* = 0.273). On the other hand, concerning the presence of neovascularization, regarding both dye leakage in FA (Figure S1D, Regression Test for Funnel Plot Asymmetry: z = −0.86, *p* = 0.391) and fluid on OCT (Figure S1E, Regression Test for Funnel Plot Asymmetry: z = −1.029, *p* = 0.304), it was not possible to identify sufficient evidence about potential publication bias. It is important to analyze these results with caution since the number of studies is relatively low, which can compromise the statistical significance of the publication bias tests.

#### 2.4.2. Safety Outcomes

Regarding the following outcomes of safety, a positive log odds ratio concerning a specific treatment, namely BVZ or RBZ, reveals a higher incidence of the respective event, meaning that it favors the other drug used.

**Figure 4:**
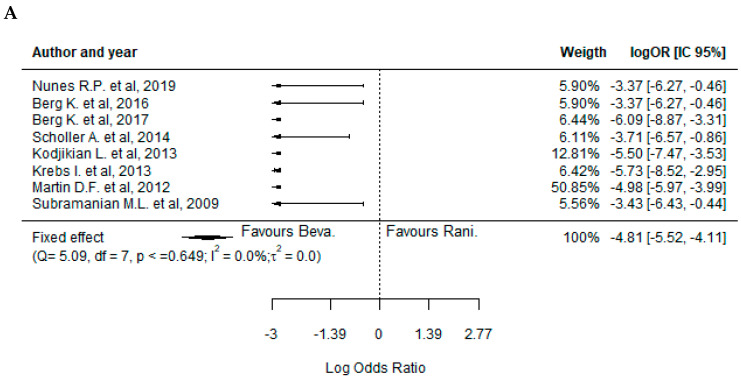

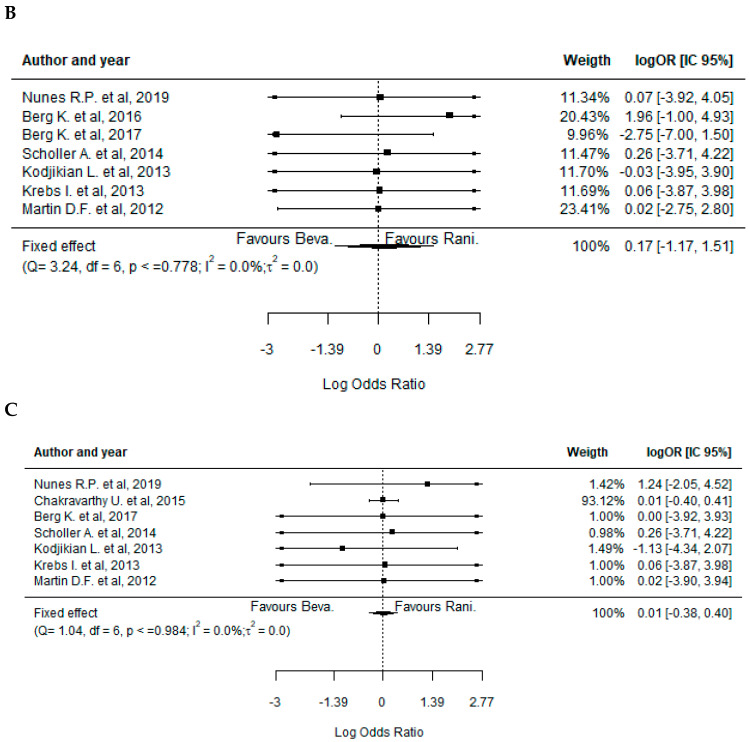
Forest plots representing the ocular safety outcomes regarding the treatment with bevacizumab and ranibizumab. Legend: (**A**) endophthalmitis; (**B**) pseudoendophthalmitis; (**C**) hemorrhage, either conjunctival or subconjunctival [28,29,31,32,34,35,36,37,39].

**Figure 5:**
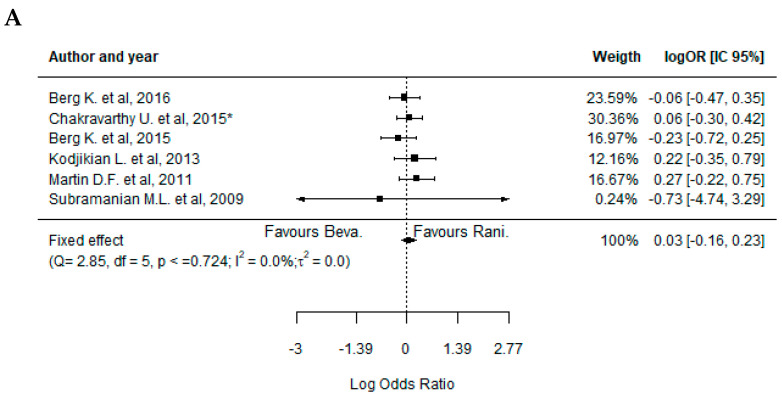

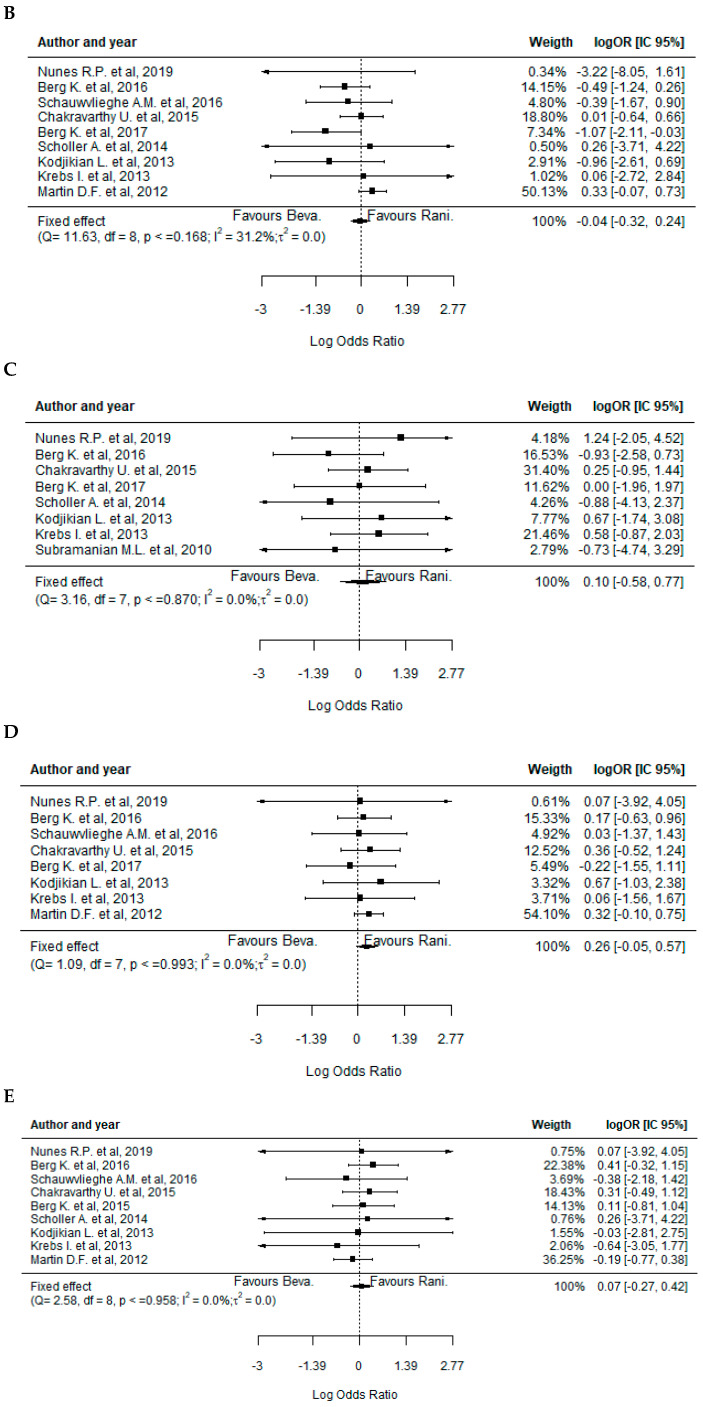
Forest plots representing the systemic safety outcomes regarding the treatment with bevacizumab and ranibizumab. Legend: (**A**) serious systemic events; (**B**) cardiovascular disorders; (**C**) vascular abnormalities; (**D**) infection; (**E**) benign or malignant neoplasm. Chakravarthy et al. (2015) [31] study was analyzed considering both 1-year and 2-year (*) treatment outcomes [28,29,30,31,32,34,35,36,37,39].

**Figure 6:**
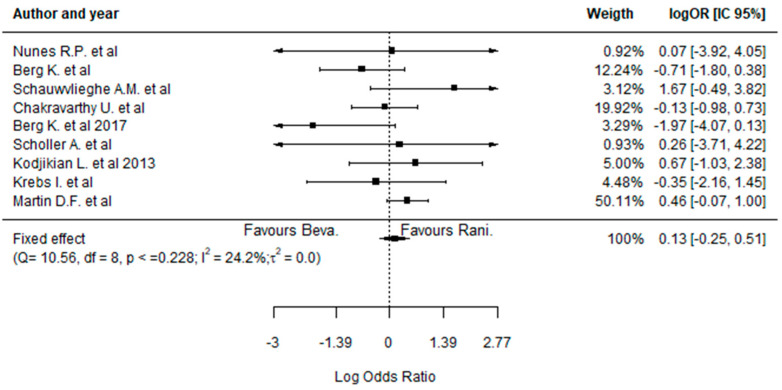
Forest plot representing the incidence of injury or procedural complications during the treatment with bevacizumab and ranibizumab [28,29,30,31,32,34,35,36,37].

Similar to the analysis of the efficacy outcomes, it was not possible to identify significant differences between BVZ- and RBZ-treated patients regarding the safety profile in the majority of the cases. In fact, the only statistically significant difference was related to a higher incidence of endophthalmitis events in the BVZ-treated patients, favoring the treatment with RBZ (Figure 4A) (−4.81, 95%CI: [−5.52; −4.11]). On the other hand, concerning the incidence of pseudoendophthalmitis (Figure 4B) (0.17, 95%CI: [−1.17; 1.51]), hemorrhage, conjunctival or subconjunctival hemorrhage (Figure 4C) (0.01, 95%CI: [−0.38; 0.40]), systemic serious events (Figure 5A) (0.03, 95%CI: [−0.16; 0.23]), cardiovascular disorders (Figure 5B) (−0.04, 95%CI: [−0.32; 0.24]), vascular abnormalities (Figure 5C) (0.10, 95%CI: [−0.58; 0.77]), infection (Figure 5D) (0.26, 95%CI: [−0.05; 0.57]), benign or malignant neoplasm (Figure 5E) (0.07, 95%CI: [−0.27; 0.42]), and injury or procedural complications (Figure 6) (0.13, 95%CI: [−0.25; 0.51]), the results were quite similar between the two drugs in all studies analyzed.

Concerning the analysis of the publication bias related to the studies analyzed (Regression Test for Funnel Plot Asymmetry), it was only possible to identify sufficient evidence in the incidence of cardiovascular disorders after the treatment with BVZ or RBZ (Figure S2A, z = −2.182, *p* = 0.029). In this sense, regarding the incidence of endophthalmitis (Figure S2B, z = 0.85, *p* = 0.395), pseudoendophthalmitis (Figure S2C, z = −1.029, *p* = 0.304), systemic serious events (Figure S2D, z = −0.202, *p* = 0.840), hemorrhage, conjunctival or subconjunctival hemorrhage (Figure S2E, z = 0.076, *p* = 0.939), vascular disorders (Figure S2F, z = −0.229, *p* = 0.819), infection (Figure S2G, z −0.402, *p* = 0.688), benign or malignant neoplasm (Figure S2H, z = −0.220, *p* = 0.826), and injury or procedural complications (Figure S2I, z = −0.775, *p* = 0.439), there was no evidence to ensure publication bias.

#### 2.4.3. Efficiency Outcome

To analyze the efficiency of each treatment, the mean number of administrations performed throughout the therapeutic regimen was considered. Theoretically, a higher number of intravitreal injections means that it is less favorable for the patients.

**Figure 7:**
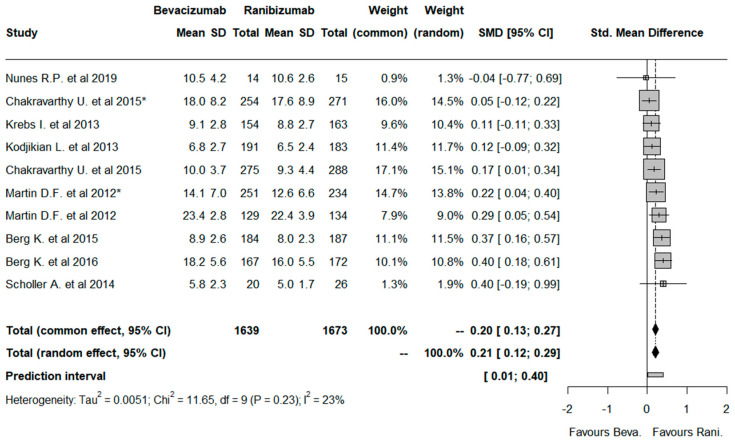
Forest plot representing the mean number of administrations regarding the treatment with bevacizumab and ranibizumab. Legend: Chakravarthy et al. (2015) [31] study was analyzed considering both 1-year and 2-year (*) treatment outcomes. Martin et al. (2012) [37] study was evaluated considering both monthly and “as needed” (*) treatment protocols [28,29,31,32,34,35,36,37].

Regarding the 10 studies analyzed, it was possible to determine a significantly higher mean number of administrations in RBZ-treated patients when compared to those treated with BVZ (Figure 7) (0.20, 95%CI: [0.13; 0.27]).

Considering the analysis of the potential publication bias, it was not possible to demonstrate sufficient evidence regarding it (Figure S3, Egger’s test: t = 4.68, *p* < 0.001).

## 3. Discussion

Globally, AMD is characterized as a major health problem considering its increased prevalence and strong trend of new cases in the future [1]. Indeed, this disease presents high direct (e.g., clinical management) and indirect (e.g., loss of productivity) costs to society [40]. Currently, the treatment of nAMD is mainly related to the intraocular administration of aVEGF molecules (e.g., RBZ, brolucizumab, aflibercept, and BVZ) and laser therapy [1]. This systematic review and meta-analysis aimed to evaluate the efficacy and safety of BVZ in patients with nAMD, along with its efficiency, when compared to RBZ. Concerning the expiring of some patents related to aVEGFs and, consecutively, a reduction of their prices in the near future, our team believed it would be pertinent to compare two of the most used aVEGFs available on the market, namely BVZ and RBZ [26].

Regarding the analysis of efficacy outcomes, it was possible to observe similar results between the treatment with BVZ and RBZ, especially considering the visual acuity and retinal and foveal thickness. Even in competition with newer molecules, such as aflibercept, the off-label use of BVZ has shown comparable efficacy to other drugs normally used, along with a lower cost for the patients [18,19,20,21]. The results obtained in this meta-analysis are in accordance with the current literature since it has been demonstrated that the treatment with BVZ is non-inferior to RBZ [30,35,41,42]. In addition, a lack of significant differences between RBZ and aflibercept has also been described in terms of changes in best-corrected visual acuity and central macular thickness [43]. However, concerning neovascularization, the treatment with BVZ was shown to be more effective than RBZ. In fact, it was shown that the treatment with BVZ, over a longer period of time, namely 24 months, provided a more extensive reduction of neovascularization (dye leakage in FA) when compared to RBZ. According to the literature, the intraocular treatment with BVZ takes a longer time to achieve the maximum effect; however, it is more stable over time compared to other pharmacological approaches, such as RBZ [44].

On the other hand, taking into consideration the safety profile of BVZ, a higher observed rate of systemic side effects would be expected in comparison to RBZ, according to other meta-analyses performed [23,24,41]. Indeed, this can be justified by the fact that BVZ has greater systemic absorption and half-life duration than RBZ [45,46]. However, considering the results obtained in this meta-analysis, it was not possible to corroborate the clinical evidence currently available since there were no significant differences between both drugs concerning the incidence of systemic side effects. Similarly, the treatment with RBZ has also been shown to be safer than BVZ regarding ocular side effects. In fact, intravitreal injections of BVZ have been associated with a higher risk of ocular inflammation when compared to RBZ [45,47]. Our results suggest that the treatment with BVZ may be associated with a higher risk of endophthalmitis than RBZ. Considering the other ocular safety outcomes, the treatment with both molecules showed comparable results. Importantly, the increase in intraocular pressure usually observed during the repetitive treatment with aVEGF agents, mainly BVZ and RBZ [48,49], should also be emphasized. However, due to the reduced number of randomized clinical trials analyzed, it was not possible to clearly analyze and compare the incidence and extension of this event between the two drugs. Furthermore, it would also be interesting to compare this safety outcome with aflibercept since it presents a reduced risk of glaucoma in patients in comparison to BVZ and RBZ [48,49].

Concerning the efficiency of BVZ, a significantly reduced number of administrations was observed when compared to the treatment with RBZ. Therefore, BVZ-treated patients usually experience fewer intravitreal injections compared to those treated with RBZ, with comparable efficacy and safety outcomes. As previously referred, BVZ presents a longer half-life than RBZ, which reduces the mean number of administrations required throughout the treatment protocol [44]. Currently, there is a reduced number of meta-analyses that evaluate this efficiency outcome, highlighting the importance of investigating it. Indeed, it is the first time, to our knowledge, that a meta-analysis has been performed considering the mean number of administrations performed in nAMD patients treated with BVZ or RBZ. Clinically, it is not possible to observe a consensus in terms of the number of required administrations between both drugs since the results are controversial among the studies in most cases [32,34,35,39]. However, the opportunity for a greater number of patients, especially in developing countries, to have access to this more economical therapeutic approach must be considered in a disease where long-term treatment resistance is prevalent [50,51].

At present, there is no cure known for nAMD, and clinical management aims to reduce its rate of progression [2,52]. The first-line medication recommended by the American Academy of Ophthalmology is the intravitreal administration of BVZ, RBZ, and aflibercept [22,53]. The majority of these aVEGF drugs are extremely costly; however, there are some substantial price differences between them. Indeed, recent evidence demonstrated that each dose of BVZ costs approximately 70.86$, which makes it less expensive when compared to RBZ (333.55$) and aflibercept (921.65$) [22]. Assuming a chronic pharmacological treatment of AMD, one of the major key factors that should be discussed is related to the direct costs for society and, more specifically, to the patient themself. Therefore, considering comparable efficacy and safety outcomes between the drugs, previously referred to as RBZ and BVZ, the price difference can be recognized as the tiebreaker factor. In addition, regarding the results from this meta-analysis, it was also shown that BVZ-treated patients experience fewer intravitreal injections than those treated with RBZ. In this sense, the treatment with BVZ presents an added therapeutic value, which is related to the lower number of administrations required and its reduced cost throughout the treatment protocol.

Despite the importance of the present systematic review and meta-analysis for the research and medical communities, there are some limitations to be aware of. Firstly, a major limitation is related to the unique comparison between BVZ and RBZ, excluding other commonly used aVEGF drugs (e.g., aflibercept and brolucizumab). Indeed, we decided not to include other pharmacological agents since RBZ is the gold standard treatment approach in nAMD [15]. Moreover, we believe that aflibercept requires an in-depth study due to its growing potential, especially with the latest 8 mg formulation. On the other hand, concerning brolucizumab, there are several reports of intraocular inflammation upon intravitreal therapy, as well as severe vision loss, making it difficult to recognize as a potential gold standard approach [54]. Nevertheless, it would be interesting to continue investigating the efficiency of BVZ compared to other aVEGF agents. In addition, the relatively low number of randomized clinical trials considered for meta-analysis concerning the eligibility criteria can also be emphasized. Furthermore, it also excluded clinical studies where a switching therapy was performed, which is a common event observed during the pharmacological treatment of nAMD. Finally, short-term clinical studies are important to evaluate the initial phase of the treatment concerning its efficacy and safety profiles; however, they were not considered for meta-analysis.

As a key strength, the use of a comprehensive and highly sensitive search strategy can be emphasized to allow the identification of high-quality randomized clinical trials. In fact, considering the studies included, the majority were qualified as high-quality, representing a reduced risk of bias. Additionally, this analysis only included studies with direct comparisons, avoiding potential heterogeneity of the results. Finally, the extensive analysis conducted regarding the efficacy, safety, and efficiency outcomes of BVZ and RBZ should also be considered. Indeed, the compilation of these data may provide a clearer vision for the medical community about the use of these two aVEGF agents, promoting more conscious decision-making.

## 4. Material and Methods

The present systematic review and meta-analysis followed the recommendations of the Preferred Reporting Items for Systematic Reviews and Meta-analysis (PRISMA) (Supplementary Material S2) [55]. In addition, this review protocol was successfully registered in the International Prospective Register of Systematic Reviews (PROSPERO, CRD42021244931) and published [6]. The PICOS strategy [(P)articipants (diagnosed with nAMD), (I)ntervention (BVZ), (C)omparison (RBZ), and (O)utcome (efficacy, efficiency, and safety) (S)tudy design (randomized clinical trials)] was used to formulate the research question: “Is BVZ more efficient than RBZ, recognized as the gold standard approach, while having comparable efficacy and safety profiles, in the treatment of patients diagnosed with nAMD?”.

### 4.1. Eligibility Criteria

Throughout the process of selection of studies, the following inclusion criteria were also taken into account: participants diagnosed with nAMD, aged 50 years and older; treatment with BVZ and RBZ, or even a placebo; presentation of the outcomes related to efficacy, safety, and/or efficiency of the treatments applied; and randomized clinical trials.

On the other hand, the following exclusion criteria were also established: participants diagnosed with polypoidal choroidal vasculopathy or retinal angiomatous proliferation, treatment switched during the study, studies written in a language other than English, and studies published before 2010.

#### 4.1.1. Participants

According to the natural history of AMD, considered by the American Academy of Ophthalmology Preferred Practice Pattern, this study only included participants diagnosed with nAMD aged 50 years and older [52].

#### 4.1.2. Intervention and Comparison

Patients diagnosed with nAMD were treated with BVZ, which was administered intravitreally in comparison to those treated with RBZ or a placebo.

#### 4.1.3. Outcomes

To evaluate the influence of intravitreal administrations of BVZ in nAMD, several outcomes were analyzed in terms of comparisons with RBZ or placebo that were related to the efficacy, safety, and efficiency profiles.

#### 4.1.4. Design

To develop this systematic review and meta-analysis, only randomized controlled clinical trials were considered.

### 4.2. Information Sources and Search Strategy

A highly sensitive search expression was developed and used in MEDLINE (24 February 2021) via PubMed platform to identify and select potentially relevant and eligible studies. The search expression used is described in the Supplementary Materials. Moreover, a filter related to publication date, excluding all articles published before 2010, was considered.

### 4.3. Selection Process

The process of screening the titles and abstracts of retrieved studies, as well as the analysis of the full text of potentially eligible articles, was developed by two independent reviewers. In case of discrepancies, a third reviewer was included to make a final decision. Inclusion and exclusion criteria were applied throughout this process to decide whether a study was eligible or not. The search results are presented in a PRISMA-adapted diagram (Figure 1), as well as the reasons for excluding the respective articles.

### 4.4. Data Collection Process

The retrieved studies from the MEDLINE database were exported and analyzed through a Systematic Reviews web application (Rayyan QCRI), which was used throughout the review for study screening and overall management. The relevant data were extracted by the same two independent reviewers and included specific parameters concerning the population, intervention, and comparison, as well as the outcomes related to the efficacy, safety, and efficiency profiles regarding the treatments applied. Once again, in case of conflict between the reviewers, a third reviewer was included to make a final decision. The included studies were descriptively analyzed and presented in a tabular form. In addition, the studies excluded from a quantitative synthesis were subjected to a qualitative analysis to withdraw all the important data available.

### 4.5. Data Items

During the process of data collection, several parameters were considered, which were, respectively, extracted and organized as follows:

- Outcomes of efficacy—visual acuity, retinal thickness, fovea thickness, and presence of neovascularization (dye leakage on FA and fluid on OCT);
- Outcomes of safety—presence of endophthalmitis; presence of pseudoendophthalmitis; serious systemic event; increased intraocular pressure, presence of hemorrhage, conjunctival or subconjunctival hemorrhage; cardiovascular disorders; vascular disorders; infection; benign or malignant neoplasm; injury or procedural complications;
- Outcomes of efficiency—number of administrations, frequency of administration, and duration of treatment.

### 4.6. Study Risk of Bias Assessment

All the selected studies were assessed by two independent reviewers to evaluate their methodological quality and potential risk of bias. In case of discrepancies between the reviewers, an additional reviewer was included to make a final decision. To evaluate the methodological quality and risk of bias, the Critical Appraisal Skills Programme Randomised Controlled Trials checklist [27] was used. Indeed, each study was scored (“Non defined”—0 points; “No”—1 point; and “Yes”—2 points) in each category present in the respective checklist, making it possible to develop an overall quality grading for each included study, which was as follows: A [18–26 points]—high quality (low risk of bias); B [9–17 points]—moderate quality (moderate risk of bias); and C [0–8 points]—low quality (high risk of bias).

### 4.7. Statistical Analysis

When data from at least 3 studies could be combined, a meta-analysis was performed to obtain a pooled estimate of effect sizes, which was analyzed using standard mean differences and odds ratios, depending on the characteristics of the outcomes under analysis. Several studies presented their results using medians and quartiles, where, in that situation, they were converted to means and standard deviations using the quantile estimation method proposed by Wan et al. (2020), where a log-normal distribution was pre-specified [56]. Either fixed-effect or random-effect meta-analyses were conducted based on an evaluation of the clinical comparability of studies and the presence of statistical heterogeneity. The decision to consider random-effect or fixed-effect models was made based on the analysis of heterogeneity among the studies, which were conducted based on the I^2^ statistic and the chi-square test (*χ*^2^). I^2^ > 50% and *p*-value < 0.2 for *χ*^2^ were considered indicative of random-effect models. We also interpreted tau^2^ (τ^2^), which provides a numerical estimate of the amount of variation in effect sizes that is beyond what would be expected due to random chance alone. If τ^2^ is close to zero, it suggests low heterogeneity among the effect sizes. We explored subgroup analyses to identify potential reasons for the observed heterogeneity whenever data were accessible (at least 2 studies per group), considering the duration of treatment of 12 months and 24 months. We also presented predictive intervals in the meta-analysis since they provide an estimate of the range of values that can be expected for the effect size of a future study, given the results of the studies included in the meta-analysis. These intervals are wider than confidence intervals, as they consider both within-study and between-study variability. Forest plots were made to show the odds ratios and standard mean differences and the corresponding 95% CI of each outcome. We conducted a sensitivity analysis to assess the impact of the risk of bias in studies, which is given in Supplementary Materials. To evaluate the potential for publication bias, we used funnel plots and Egger’s regression test or Regression Test for Funnel Plot Asymmetry. The level of significance was set at 0.05, and statistical analysis was performed using R statistical software (version 4.3.2.), and meta and metafor packages were used.

## 5. Conclusions

This systematic review and meta-analysis revealed comparable efficacy and safety profiles between BVZ and RBZ. However, regarding the efficiency of BVZ, the results suggested that patients needed a reduced number of administrations compared to those treated with RBZ. In this sense, the data obtained in this study provides valuable evidence for both research and medical communities, highlighting the off-label use of BVZ in the clinical management of nAMD.

## Data Availability

All data created throughout the project is present in the manuscript and Supplementary Materials. Further information can be obtained by contacting the corresponding author.

## Supplementary Materials

The following supporting information can be downloaded at https://www.mdpi.com/article/10.3390/ph17081000/s1. The supporting data, namely the search expression used and the figures referred to in the Results section, are present in the Supplementary Materials files. Figure S1: Funnel plots regarding the publication bias assessment in the efficacy outcomes evaluated. Legend: A, visual acuity; B, retinal thickness; C, fovea thickness; D, dye leakage in FA; E, fluid on OCT; Figure S2: Funnel plots regarding the publication bias assessment in the safety outcomes evaluated. Legend: A, cardiovascular disorders; B, endophthalmitis; C, pseudoendophthalmitis; D, systemic serious events; E, hemorrhage, conjunctival or subconjunctival hemorrhage; F, vascular disorders; G, infection; H, benign or malignant neoplasm; I, injury or procedural complications; Figure S3: Funnel plot regarding the publication bias assessment in the efficiency outcome evaluated. Reference [55] is cited in the supplementary materials.
