# Supplementary material for: Off-Label Use of Bevacizumab in Patients Diagnosed with Age-Related Macular Degeneration: A Systematic Review and Meta-Analysis
Source: Pharmaceuticals (Basel). 2024 Jul 29;17(8):1000. doi: 10.3390/ph17081000 (PMC11357420; doi:10.3390/ph17081000)
Supplement: Supplementary file 1 [file pharmaceuticals-17-01000-s001.zip › Figures S1-S3.pdf]

Search expression:

((("Middle Aged"[Mesh] OR “Middle Aged”[tiab] OR “Middle Aged” OR "Aged"[Mesh] OR Aged[tiab] OR Aged OR Elderly[tiab] OR Elderly OR Elder) AND ("Wet Macular Degeneration"[Mesh] OR “Wet Macular Degeneration”[tiab] OR “Wet Macular Degeneration” OR “Macular degeneration”[tiab] OR “Macular degeneration” OR AMD[tiab] OR “Neovascular Age-Related Macular Degeneration”[tiab] OR “Neovascular Age-Related Macular Degeneration” OR “Age-Related Macular Degeneration”[tiab] OR “Age-Related Macular Degeneration”)) AND (“Bevacizumab”[Mesh] OR Bevacizumab[tiab] OR Bevacizumab))

Supplementary Figures

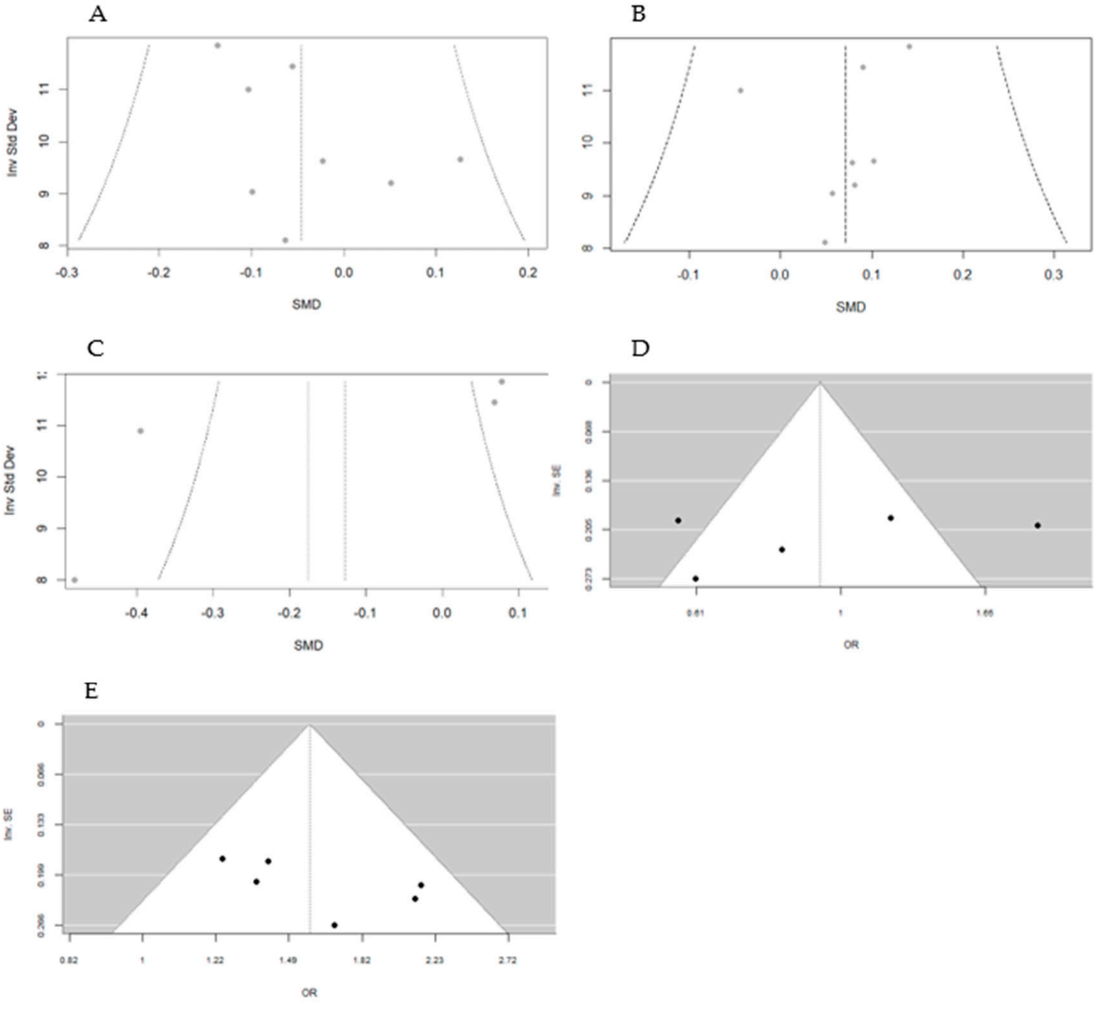

Figure S1 – Funnel plots regarding the publication bias assessment in the efficacy outcomes evaluated. Legend: A, visual acuity; B, retinal thickness; C, fovea thickness; D, dye leakage in FA; E, fluid on OCT.

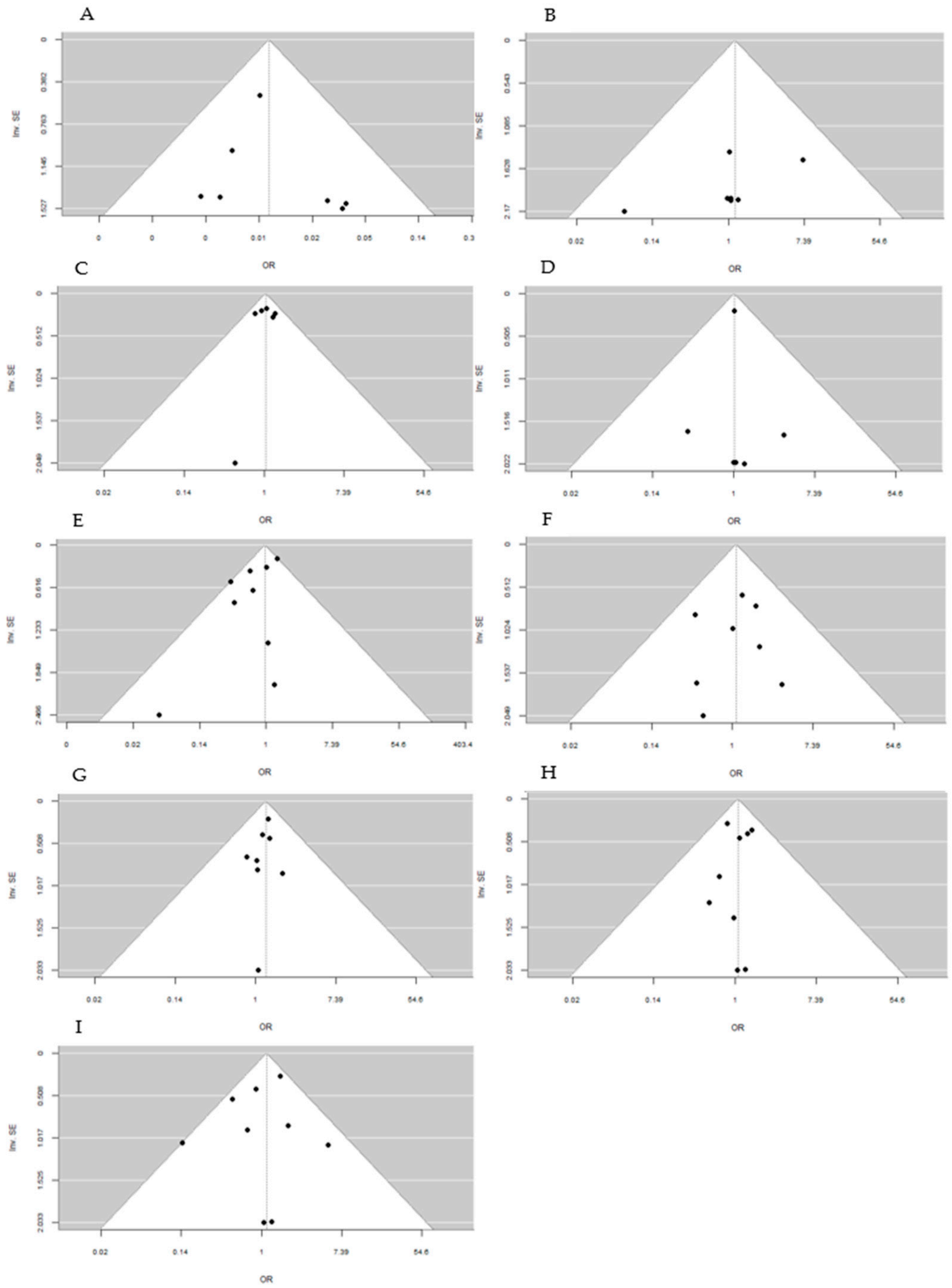

Figure S2 – Funnel plots regarding the publication bias assessment in the safety outcomes evaluated. Legend: A, cardiovascular disorders; B, endophthalmitis; C, pseudoendophthalmitis; D, systemic serious events; E, hemorrhage, conjunctival or subconjunctival hemorrhage; F, vascular disorders; G, infection; H, benign or malignant neoplasm; I, injury or procedural complications.

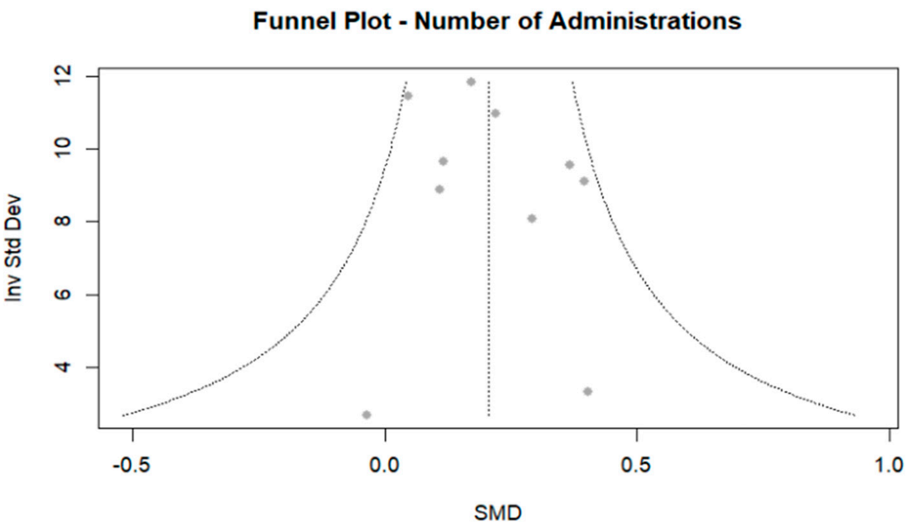

Figure S3 – Funnel plot regarding the publication bias assessment in the efficiency outcome evaluated.
